# Assessment of data quality in an international multi-centre randomised trial of coronary artery surgery

**DOI:** 10.1186/1745-6215-12-212

**Published:** 2011-09-26

**Authors:** Lukasz J Krzych, Belinda Lees, Fiona Nugara, Winston Banya, Andrzej Bochenek, Jo Cook, David Taggart, Marcus D Flather

**Affiliations:** 1 Clinical Trials and Evaluation Unit, Royal Brompton and Harefield NHS Foundation Trust, London, UK; 2 1st Department of Cardiac Surgery, Medical University of Silesia, 47 Ziolowa Street, 40635 Katowice, Poland; 3 John Radcliffe Hospital, University of Oxford, Oxford, UK

## Abstract

**Background:**

ART is a multi-centre randomised trial of cardiac surgery which provided a unique opportunity to evaluate the data from a large number of centres from a variety of countries. We attempted to assess data quality, including recruitment rates, timeliness and completeness of the data obtained from the centres in different socio-economic strata.

**Methods:**

The analysis was based on the 2-page CRF completed at the 6 week follow-up. CRF pages were categorised into "clean" (no edit query) and "dirty" (any incomplete, inconsistent or illegible data). The timelines were assessed on the basis of the time interval from the visit and receipt of complete CRF. Data quality was defined as the number of data queries (in percent) and time delay (in days) between visit and receipt of correct data. Analyses were stratified according to the World Bank definitions into: "Developing" countries (Poland, Brazil and India) and "Developed" (Italy, UK, Austria and Australia).

**Results:**

There were 18 centres in the "Developed" and 10 centres in the "Developing" countries. The rate of enrolment did not differ significantly by economic level ("Developing":4.1 persons/month, "Developed":3.7 persons/month). The time interval for the receipt of data was longer for "Developing" countries (median:37 days) compared to "Developed" ones (median:11 days) (p < 0.001). The median number of data queries was 23% in "Developed" countries compared to 19% in "Developing" ones (p = ns).

**Conclusions:**

In this study we showed that data quality was comparable between centres from "Developed" and "Developing" countries. Data was received in a less timely fashion from Developing countries and appropriate systems should be instigated to minimize any delays. Close attention should be paid to the training of centres and to the central management of data quality.

**Trial registration:**

ISRCTN46552265

## Background

International multi-centre randomised trials are widely used to evaluate new investigational medicinal products or treatment strategies. It is essential that only accurate and verified data are collected in these trials in order that the results are reliable particularly as this may be used to inform guidance and recommendations for everyday clinical practise. However the collection of high quality data in these trials can be challenging because of several potential difficulties e.g. the inclusion of multiple centres with different research experience, different cultures and healthcare systems, language difficulties, and the sheer number of people involved in collecting and sending data.

Quality assurance is the key point in all steps of data management, beginning with data generation and entering data on to case report forms (CRF) by centres, and ending with statistical analysis and presentation of the results [1]. Data quality can be variable and the purpose of quality assurance is not only to ensure that all data are correct but also to ensure that any observed treatment effects are authentic and their estimated magnitude is unbiased so that clinical trial results are reliable. Inappropriate CRF or questionnaire management may produce bias and a lack of precision in the estimates of treatment effects [2]. Therefore quality assurance is a cornerstone in improving data quality [3-5].

Even when systematically controlled, databases in clinical trials may include errors. For example in a multi-centre clinical trial comparing methods of treatment of uterine cervical cancer a data accuracy of 81.8% was found and both problems in data management but also a lack of clarity of the CRF were to blame [6]. Nahm et al. revealed that the average error rate for published CRF-to-database comparison audits was on average 14.3 per 10, 000 fields [7]. The issue was also described in cardiac surgery studies [8,9]. However, often these errors have been described in registries rather than from randomised clinical trials.

The Arterial Revascularisation Trial (ART) is an international multi-centre randomised clinical trial designed to compare single internal mammary artery (IMA) with bilateral IMA grafting in patients undergoing coronary artery by-pass graft (CABG) surgery [10]. Since ART is one of the largest cardiac surgery trials ever to be undertaken it provides a unique opportunity to evaluate the data from a large number of centres from a variety of countries with different socio-economic status and to perform a systematic analysis of the quality of the data from the different centres.

Our main aim was to compare the data quality obtained from the centres in different socio-economic strata. We wanted to compare the following:

1. Recruitment rates across different sites and relate this to socio-economic status

2. Time differences for receipt of data at 6 weeks follow-up

3. Completeness of the data assessed by the number of data queries

Our hypothesis was that neither recruitment rates nor data quality and time delay in sending the data are dependent on the socio-economic status of the country of the participating site.

## Methods

ART is a multi-centre two-arm randomised trial designed to determine if the use of both mammary arteries during CABG surgery improves survival, and reduces the chance of recurrent angina and/or the need for further intervention (including further cardiac surgery or percutaneous coronary intervention) compared to using one mammary artery. CABG patients with multi-vessel coronary artery disease were considered for inclusion into the study. The exclusion criteria were as follows: single graft, redo-CABG, evolving myocardial infarction and concomitant valve surgery. After giving written informed consent patients were randomised into the trial. Patients were followed up at 6 weeks post surgery and then annually for up to 10 years. The main outcome is survival but patients are also being followed up for myocardial infarctions, angina symptoms, strokes or any other clinical adverse events [10].

ART is supported by grants from the Medical Research Council (MRC) and the British Heart Foundation (BHF). In the original funding application to the MRC and BHF, centres from the UK, Italy and Australia were identified as potential centres. However, once the study was underway, other centres from Austria, Poland, Brazil and India also expressed an interest in participating.

All centres in ART received a training visit from a member of the co-ordinating centre (CTEU, Royal Brompton Hospital, London, UK) where the requirements for data collection, completion of the CRFs and management of the data were described in a standardised format. These visits ensured that the investigators at each site (including principal investigator, co-investigators and co-ordinators) fully understood the Protocol and the practical procedures for the study described in the Manual of Operations and the importance of conducting the study to Good Clinical Practice (GCP). Study site co-ordinators were responsible for gathering and recording data, and handling and resolving any edit queries.

Data collection in ART is based on a paper system with central monitoring of the data. A two-part no-carbon required (NCR) CRF was created to collect baseline, in-hospital surgical information and follow-up data. The participating centres were required to complete the relevant CRF pages, tear off the top copy and then send these pages to the CTEU by post or fax within the obligatory timelines (Table 1). On receipt of these data, the CTEU would review and log all data into the database in the first instance. Data would then be entered into a bespoke database system. If any inconsistent, missing, or illegible data were found, a data query would be raised. Each data query would request clarification of either one or more data points. Each query would be sent by fax to the centre for resolution. The participating centres were given a deadline of 3 weeks to return the corrected data by fax to CTEU. In the event of not receiving this information, centres would be sent a reminder to send these data. On receipt of the corrected information, the CTEU would then update the database with the appropriate information and then the query would be closed.

**Table 1:** Trial phases and time frame for receipt of study documentation

Trial Documents	Time Frame
Screening data	Mailed to CTEU within 17 days of randomisation
In-hospital data	Mailed to CTEU within 17 days of the surgery
6 week follow-up	Mailed to CTEU within 17 days of follow up visit
Annual follow-ups	Mailed to CTEU within 17 days of follow up visit
Edit queries	Faxed to CTEU within 3 weeks of receipt
Adverse event form	Faxed to CTEU within 72 hours of knowledge of the event

As described above, our main hypothesis was that neither recruitment rates nor data quality and time delay in sending the data are dependent on the socio-economic status of the country of the participating site. In this observational study, to test the hypothesis we performed an analysis based on the 2 page CRF that should be completed at the 6 week follow-up. Overdue 6 week data would be chased at 60 days post randomisation (42 days + 17 days for completion and postage of the CRF pages to the CTEU. The data-points from the 6 week follow-up CRF pages formed the basis for the assessment of the data query generation and are shown in Additional file 1. CRF pages were reviewed and categorised into "clean" and "dirty". A clean CRF was classified as one with no edit queries on first receipt. Each CRF page was classified separately. Each variable from the two pages was categorised into either "no edit query raised", or "edit query raised". If any data were incomplete, inconsistent or illegible, CTEU raised a data query requesting the centre to clarify the data. The timelines were assessed on the basis of the time interval from the 6 week follow-up visit and receipt of complete (verified) CRF at CTEU (Table 2).

**Table 2:** Timelines established to assess time gap between 6-week visit and receipt of data

'CLEAN' DATA			'DIRTY' DATA
Randomisation			Randomisation
**I**			**I**
Surgery			Surgery
**I**			**I**
6 week follow-up visit	TIME GAP	TIME GAP	6 week follow-up visit
**I**			**I**
Data received			Data received
**I**			**I**
Data entered into a database			Data sent back for correction
			**I**
			Corrected data received
			**I**
			Data entered into a database

The number of data queries raised per patient (counting a maximum of one data query per CRF variable) was counted. The percentage of data queries per patient was then calculated based on the number of 42 possible queries to be generated in total (see Additional file 1). CRFs for all patients were analysed and presented in the results. The number of recruited participants was established on the basis of the date of first patient enrolled as the reference date. Only whole months of enrolment in the analysis were included. Rate of recruitment were expressed as the number of patients enrolled per month.

### Statistical analysis

Our primary goal was a comparison of recruitment rates and data quality between countries. Data quality was defined as the number of data queries (in percent) and time delay (in days) between 6 week follow-up visit and receipt of correct data. Analyses were stratified on the basis of the socio-economic level into two categories: "Developing" countries (Poland, Brazil and India) and "Developed" (Italy, UK, Austria and Australia), according to the World Bank data [11]. We also assessed the impact of enrolment on the number of data queries and time elapsed between 6-week visit and receipt of data.

Variables are shown as arithmetic mean and standard deviation (for normally distributed quantitative variables) or median (Me) and interquartile range (IQR) (for non-normally distributed quantitative data), or percent (for qualitative data). Correlation between quantitative variables was determined on the basis on Spearman rank coefficients. Between-group comparisons were performed using Mann-Whitney U-test. Normality of distribution for continuous data was verified by Shapiro-Wilk W-test. Non-normally distributed data underwent logarithmic transformation before further analyses. 'P' value < 0.05 was considered statistically significant.

## Results

In the ART trial 3102 patients were randomised within 28 centres in 7 countries over 42 months. There were 18 centres (with 2326 randomised patients) in the "Developed" and 10 centres (with 676 randomised patients) in the "Developing" countries.

The total recruitment period was 42 months. Only 6 centres recruited patients for 3 years or more. The median number of months for recruitment was 28 per centre (minimum 3, maximum 42) (32 for 'Developed' and 23 for 'Developing'; p < 0.001). The median recruitment by centre was 94 patients (minimum 6, maximum 427) with no significant difference between "Developed" and "Developing" countries (96 patients and 78 patients, respectively).

**Figure 1:**
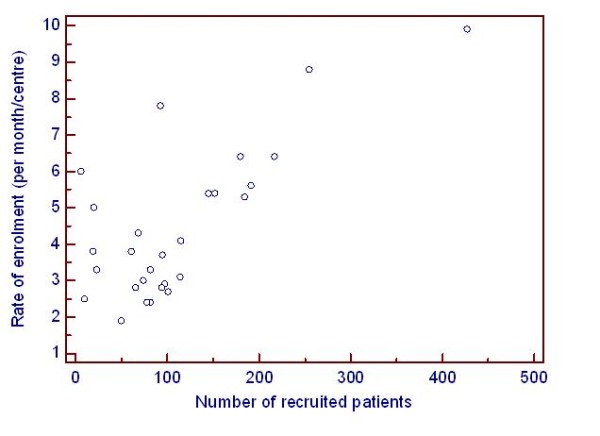
**Scatter diagram**. Scatter diagram for correlation between the rate of enrolment and the number of recruited patients by country.

The overall recruitment rate was 4.4 patients per month per centre (minimum 1.8, maximum 12.1). There was a correlation between rate of enrolment and number of recruited patients by centres in the participating countries (R^2 ^= 0.53, p < 0.001) (Figure 1).

The median time interval from 6 week follow-up visit and receipt of complete CRF was 14 days (IQR: 7, 34) and the median percent of data queries was 21% (IQR: 5, 48).

We found no correlation between the median time elapsed between 6-week visit and receipt of data, and the number of recruited patients by country (R^2 ^= 0.003, p = ns). There was also no correlation between the median percent of data queries per country and the number of recruited patients (R^2 ^= 0.04, p = ns). Finally, there was no correlation between the median percent of data queries and the median time elapsed between 6-week visit and receipt of data by country (R^2 ^= 0.02, p = ns).

**Figure 2:**
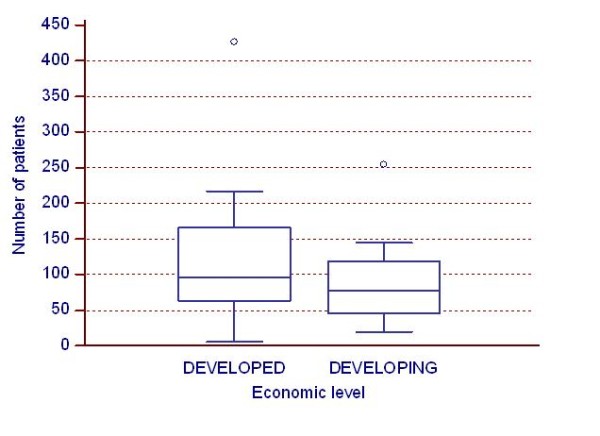
**Impact of economic level**. Impact of economic level on the number of recruited patients per country.

**Figure 3:**
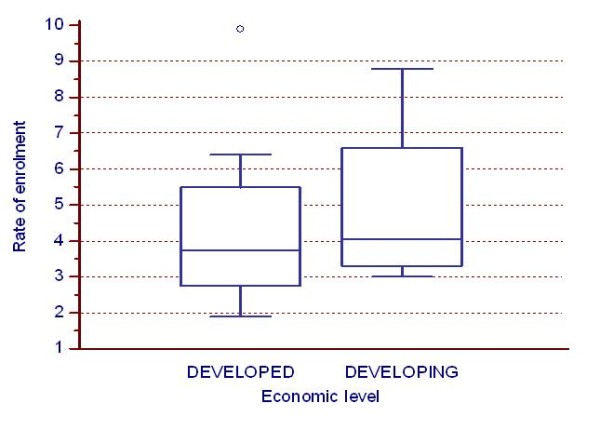
**Impact of economic level on the rate of enrolment**. Impact of economic level on the rate of enrolment (per month/centre) per country.

The number of recruited patients did not differ statistically significantly by economic level ("Developing" countries median: 83 persons/country, "Developed" countries median: 98 persons/country) (Figure 2) as well as the rate of enrolment ("Developing" countries median: 4.1 persons/month, "Developed" countries median: 3.7 persons/month) (Figure 3).

**Figure 4:**
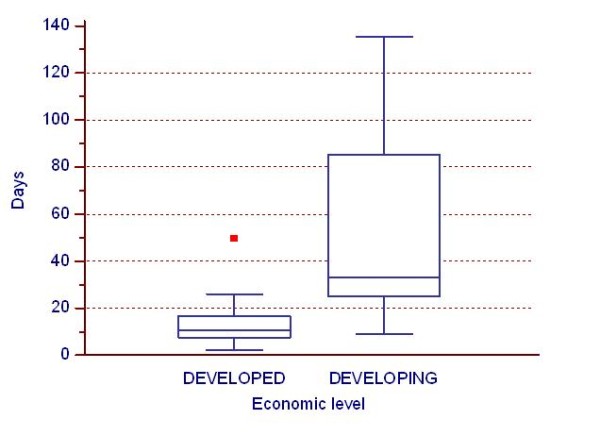
**Impact of economic level on time elapsed**. Impact of economic level on time elapsed (in days) between 6-week visit and receipt of data per country.

The time elapsed between 6-week visit and receipt of data per country by economic level in shown in Figure 4. Time interval was significantly longer for "Developing" countries (median: 37 days) compared to "Developed" ones (median: 11 days) (p < 0.001).

**Figure 5:**
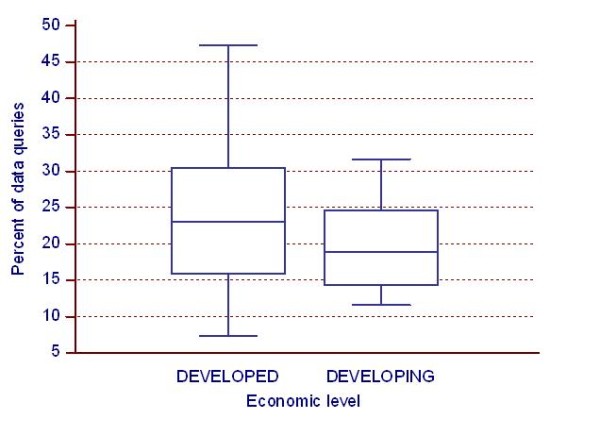
**Impact of economic level on the percent of data queries**. Impact of economic level on the percent of data queries in a 6-week follow-up visit per country. values are Me, IQR (box), range (whiskers), and extreme values (dots).

The percent of data queries in a 6-week follow-up visit per country was higher in "Developed" countries (median: 23%) compared to "Developing" ones (median: 19%) but the difference was not significant (Figure 5) (p = ns).

## Discussion

The socioeconomic status of the country did not appear to influence the numbers of patients recruited or the rate of recruitment. The timeliness of the data was slower from "Developing" countries rather than "Developed" and did not seem to affect the number of edit queries and the number of patients enrolled does not seem to affect the number of edit queries or the timeliness of the data. Those centres with the highest rate of enrolment were those who enrolled the most number of patients.

The data from this study provide some reassurance to those designing and managing multi centre trials that using a wide variety of centres with different socioeconomic status does not appear to adversely affect the quality of data as assessed by the number of data queries. The inclusion of multiple centres worldwide provides a number of advantages, in particular ensuring study recruitment is completed on time and also allowing the findings of the study to be applicable to future patients worldwide. However, there are a number of potential challenges to consider when including centres worldwide. These are cultural differences, and the variability of resources to carry out both the surgery and completion of study related tasks including completing the CRFs and following patients up properly. In ART we also have to consider communication issues both between the co-ordinating centre and the participating centres and also between the centres and the patients including reliability of postal systems and access to telephones for follow-up. Additionally centres need to follow up patients if they are admitted to other hospitals and the systems to do this and obtain the necessary medical summaries are variable.

We searched Medline for other clinical trials that had evaluated data quality and found three trials although none were exactly related to our analyses. One of these trials was an oncologic international trial conducted in the Netherlands and Indonesia. The authors showed that using an electronic medical records system helped to reduce data error rates, especially those critical for the primary goals of the trial [12]. They also found that during the study period the quality of data improved. Out of 433 CRFs submitted for the first time 33.7% needed some corrections but none of them had more than 2 errors in the primary data. Five months after the start of study the error rate for the primary data items was just 1.6%. It needs to be clarified that the analysis included only 2 countries so generalisation of its findings is limited.

In the second study, Tolmie and colleagues assessed the data quality submitted to the Clinical Endpoint Committee for adjudication [13]. They assessed the information submitted in the packages to the Committee for the endpoint events from 25 countries. Data quality was rather poor. They found that 782 queries were generated in 1595 endpoint packages reviewed amongst which 78.9% generated only one query. Interestingly, no source data queries were generated for countries with no more than 25 recruited subjects, but both low recruiting and high recruiting countries had a high number of queries relating to subject identifiers. The time between the query being submitted to the sponsor and being resolved ranged from one day to 22.8 weeks (Median 23, IQR 1.61) [13].

In the third study, the Type 1 Diabetes Genetics Consortium Trial, the authors reported good data quality with a low percentage of missing data and low duplicate data entry error rate (up to 0.5%) [14]. Using an electronic data entry system they found some differences in data collection between 214 participating centres. The highest rate of errors was found for Asia-Pacific countries and the United Kingdom, and the lowest was in European and North American centres.

To address the potential challenges of involving multiple centres worldwide Aitken et al. suggested multidimensional strategies are used to administer such a trial. They found that the approaches include using experienced project coordinators, increasing communication between centres, implementation of strategies to optimise intervention compliance, site-specific recruitment and retention techniques, centralisation of data management and consideration of ethical and budgetary requirements at local sites [15]. Frank et al. recommended that to ensure high recruitment goals and high quality of study it is necessary to have bilingual investigators and staff members who spent time at one another's sites, make use of frequent conference-call staff meetings and be flexible within the bounds of the sometimes contradictory requirements of the local authorities [16]. At a site level a collaborative relationship between researcher and practice staff is an important issue for recruitment and retention, and for data quality [17]. The factors responsible for good performance of the trial are also study leadership and experience of clinical centre staff. So establishing an organizational structure that provide leadership, site-to-site communication, understandable performance criteria, a proper process for data monitoring and providing feedback may guarantee success of the clinical trial [18].

There are some limitations of our current study. First, we use a paper-based system to complete data and to validate their quality. Medical record abstraction is the most significant source of errors and should be measured and managed appropriately and in a timely fashion during the course of the trial. Researchers and co-ordinating centres are transitioning from paper systems to electronic data capture which are successfully integrated into clinical practise and are believed to be of higher quality compared with paper based systems [19]. Moreover there are many attempts to quantify data quality for clinical trials using electronic data collection that give reproducible quality control and make trials more valid and scientifically stringent [5,7,15,20]. At the co-ordinating centre for ART, the CTEU performed central monitoring of data to ensure consistency and completeness of dataset. Second, in our analysis we grouped centres by country and then by socioeconomic status without focusing on data on individual centres which may have masked wide variation in data quality within countries. However this method was used intentionally to guarantee anonymity between centres. Finally, this paper focuses only on 6 week data which is close to the date of surgery. On the one hand, it is convenient to eliminate difficulties with patient follow up (i.e. bias caused by lost-to-follow-up). But on the other hand we cannot exclude that annual follow up may provide different results, and this should be investigated in further studies.

## Conclusions

This study provides evidence that in a large multi-centre trial, rates of recruitment, total recruitment and data quality can be comparable between centres from "developed" and "developing countries". Close attention should be paid to the training of centres and to the central management of data quality. Data may be received in a less timely fashion from developing countries and appropriate systems should be instigated to minimize any delays. Achieving accurate and timely data is an essential step in the good conduct of a clinical trial.

## Competing interests

The authors declare that they have no competing interests.

## Authors' contributions

LJK was involved in conception and design, performed data analysis, participated in data interpretation and manuscript preparation. BL participated in conception and design, acquisition of funding, data interpretation and manuscript preparation. FN was responsible for data acquisition and manuscript preparation. WB was responsible for data analysis and data interpretation. AB participated in data interpretation. MF was involved in conception and design and data interpretation. DT participated in conception and design and data interpretation. All authors read, revised and approved the final manuscript.

## Supplementary Material

Additional file 1 Data points from the 6 week visit CRF to be used in the edit query analysis Click here for file
