# Supplementary material for: Assessment of data quality in an international multi-centre randomised trial of coronary artery surgery
Source: Trials. 2011 Sep 26;12:212. doi: 10.1186/1745-6215-12-212 (PMC3205027; doi:10.1186/1745-6215-12-212)
Supplement: Additional file 1 — Data points from the 6 week visit CRF to be used in the edit query analysis [file 1745-6215-12-212-S1.PDF]

**Appendix 1. Data points from the 6 week visit CRF to be used in the edit query analysis**

| CRF page number | Item | Item description |
|---|---|---|
| 11 | 1 | Date of assessment |
| | 2 | NYHA class |
| | 3 | CCS class |
| | 4 | Current employment status |
| | 5 | Smoking status |
| | 6 | Atrial fibrillation |
| | 7 | Sternal wound infection |
| | 8 | Sternal wound dehiscence |
| | 9 | Did infection require (Antibiotics/Debridement/Vac dressing/ reconstruction) |
| | 10 | Death |
| | 11 | Myocardial infarction |
| | 12 | Cerebrovascular accident |
| | 13 | Major bleed |
| | 14 | CABG/PTCA |
| | 15 | Other Serious Adverse Event |
| | 16 | Number of visits to GP |
| | 17 | Number of visits to nurse |
| | 18 | Number of visits to out-patient clinic |
| | 19 | Number of visits to cardiac rehabilitation clinic |
| | 20 | Number of admissions to hospital |
| | 21 | Total number of nights in hospital |
| 12 | 1 | Aspirin |
| | 2 | Clopidogrel |
| | 3 | GPIIa/IIIb agent |
| | 4 | Warfarin |
| | 5 | Other anticoagulant |
| | 6 | Beta blockers |
| | 7 | Calcium channel antagonists |
| | 8 | Nitrates |
| | 9 | Potassium channel activators |
| | 10 | Lipid lowering agent |
| | 11 | ACE inhibitors |
| | 12 | Angiotensin-II antagonists |
| | 13 | Diuretics |
| | 14 | Digoxin |
| | 15 | Amiodarone |
| | 16 | Any other cardiovascular medication |
| | 17 | Any other cardiovascular medication |
| | 18 | 1 year follow-up scheduled for |
| | 19 | Name of person completing form |
| | 20 | Signature of person completing form |
| | 21 | Date form completed |
